# Artificial intelligence and editorial process in CSP

**DOI:** 10.1590/0102-311XEN189024

**Published:** 2024-11-25

**Authors:** Luciana Correia Alves, Luciana Dias de Lima, Marilia Sá Carvalho

**Affiliations:** 1 Instituto de Filosofia e Ciências Humanas, Universidade Estadual de Campinas, Campinas, Brasil.; 2 Escola Nacional de Saúde Pública Sergio Arouca, Fundação Oswaldo Cruz, Rio de Janeiro, Brasil.; 3 Programa de Computação Científica, Fundação Oswaldo Cruz, Rio de Janeiro, Brasil.

Throughout history, technological advances have always been followed by uncertainties, as occurred during the Industrial Revolution with the emergence of machines in the late 18th century and in computing with the development of the 86-DOS system in the 1980s. In Art, the development of photography in the 19th century inspired painters such as Monet and Renoir to create Impressionism, incorporating new creative elements ^1^. While the human ability to imagine and create is essential, innovations often raise concerns about the potential for replacing human cognition.

More than 70 years have passed since the emergence of the first computers, and the technology surrounding them has advanced by leaps and bounds. With the evolution of artificial intelligence (AI), new fields and sub-areas have gained prominence, especially from November 2022, when generative AI platforms, including large language models (LLMs) such as the popular ChatGPT, have become widely used.

In this editorial, we do not intend defend the use of LLMs, but we cannot ignore the impact of these technologies in the current scenario, especially regarding their relation with the editorial process and the universe of scientific publications.

Historically, a variety of AI tools have been used by authors and journals to improve writing, correct spelling and grammar issues, generate images, detect plagiarism, and translate texts, as is the case with Google Translate, Grammarly, Hemingway Editor, Jasper AI, among others ^2^. With the increasing expansion of these platforms, especially among authors, it is up to us, editors and the publishing market, to reflect on the responsible use of these tools, aiming to optimize the scientific publication process ^3^. After all, the problem does not lie in the technology itself, but in the way it is used ^1^.

*Cadernos de Saúde Pública* (CSP) recognizes the need to keep up with the new technological context and contribute to the debate and regulation of the use of AI in the publishing process. We know that ChatGPT is being widely used as an auxiliary tool for academic writing. In this context, our goal is to understand and guide the use of these resources in a transparent and ethical manner, both by authors and by reviewers and editors. This stance is aligned CSP’s practice, which is based on the principles and guidelines of research integrity recommended by the Committee on Publication Ethics (COPE) ^4^.

When it comes to the work of reviewers and editors, AI can support many aspects of the publishing process. AI tools may be able to identify, with greater accuracy than current solutions, texts generated by LLMs compared to those written by humans, and publishers can benefit from these tools to maintain scientific integrity and rigor. Authors whose primary language is not English, for example, are often guided to seek native reviewers to improve language and grammar. However, journals could suggest the use of generative AI for this purpose, especially in simpler cases ^2^.

Furthermore, AI can be employed to create higher-quality abstracts, a practice that editors may encourage, as long as these processes are properly credited in the article by all parties involved ^2^. At an earlier stage of the article flow in the journal, editors can use these tools to make a preliminary evaluation of the manuscript, seeking to identify whether the topic has already been excessively addressed; to identify plagiarism and conflicts of interest; to locate reviewers specialized in the topic; or to edit the version accepted for publication in order to meet the journal’s standards regarding style and language ^5^ ^,^ ^6^. At the process of standardization and formatting of the article, such as adapting graphs and tables, AI can minimize staff time and costs dedicated to this task, enabling journals with fewer resources to optimize their spending and extend their longevity.

CSP considers it acceptable for authors to use ChatGPT to assist in structuring themes and topics of an article, working as a draft, suggesting titles that may attract readers’ attention or phrases more appropriate to formal language, producing and correcting programming codes, assisting in statistical analysis, or formatting the article and references according to a given standard. Generative AI can also translate texts into another language, facilitating communication between authors and their readers. Note that the authors should indicate, in the proper sections of the article (such as Methods or Acknowledgments), in which stages the tool was used. These clear strategies promote transparency in the use of technology.

COPE argues that AI tools such as ChatGPT cannot be credited as authors of scientific articles, as they cannot take responsibility for the content and neither claim copyright ^4^. CSP endorse such statement, since authors must approve the submitted final version, ensure its integrity, and sign copyright grant terms, which AI cannot do.

Moreover, the writing of scientific articles requires originality, something impossible for ChatGPT, since the texts it generates are only a synthesis of the most recurrent information regarding the subject requested in the universe of the data used in its training. This increases the risk of plagiarism or high similarity between texts. AI can generate very similar content for different articles, even with variations in form and style, without proper crediting sources, which would be easily detected by similarity checking software. AI-generated content is also often associated with copyright infringement ^2^. Biased responses, limited originality, and the potential to disseminate incorrect information are among the main problems arising from generative AI, since the results have a reduced reliability ^7^.

Usages to artificial intelligence have been widespread for years, even before they were popularly known by this name, so that they have already reached a state of ubiquity in the daily lives of most people. Examples of this are the spell checkers of text editors, the content assistants of streaming services, and the spam filters in emails. It is not surprising that people linked to scientific research and innovation are more likely to experience, in a conscious and critical way, the possibilities of using these technologies.

Scientific publishing must keep pace with contemporary transformations. With strict oversight and clear well-defined regulation, the use of AI in the editorial process can bring great benefits. Recognizing the possibilities that this technology offers already represents a significant advance. The great current challenge for all journals is to find ways to integrate, in a practical, efficient, and ethical way, some of the tasks performed by generative AI in the daily routine of the editorial process. Developing best practices and strategies for a harmonious relationship between AI, journals, and scientific publishing may be the way forward, bringing benefits to both science and society as a whole.
