# Supplementary material for: Artificial intelligence and editorial process in CSP
Source: Cad Saude Publica. 2024 Nov 25;40(11):e00189024. doi: 10.1590/0102-311XEN189024 (PMC11654109; doi:10.1590/0102-311XEN189024)
Supplement: Translation

# Translation: Inteligência artificial e processo editorial em CSP

**DOI:** 10.1590/0102-311XPT189024

**Authors:** Luciana Correia Alves, Luciana Dias de Lima, Marilia Sá Carvalho

**Affiliations:** 1 Instituto de Filosofia e Ciências Humanas, Universidade Estadual de Campinas, Campinas, Brasil.; 2 Escola Nacional de Saúde Pública Sergio Arouca, Fundação Oswaldo Cruz, Rio de Janeiro, Brasil.; 3 Programa de Computação Científica, Fundação Oswaldo Cruz, Rio de Janeiro, Brasil.

Ao longo da história, avanços tecnológicos sempre vieram acompanhados de incertezas, como ocorreu na Revolução Industrial com o surgimento das máquinas na segunda metade do século XVIII, e na computação com o desenvolvimento do sistema 86-DOS nos anos 1980. Na arte, a fotografia no século XIX inspirou pintores como Monet e Renoir a desenvolverem o impressionismo, incorporando novos elementos criativos ^1^. Embora a capacidade humana de imaginar e criar seja essencial, as inovações frequentemente levantam preocupações sobre o potencial de substituição da cognição humana.

Já se passaram mais de 70 anos desde o surgimento dos primeiros computadores, e a tecnologia em torno deles tem avançado a passos largos. Com a evolução da inteligência artificial (IA), novos campos e subáreas ganharam destaque, especialmente a partir de novembro de 2022, quando plataformas de IA generativa, incluindo modelos de linguagem natural (LLM), como o popular ChatGPT, passaram a ser amplamente utilizadas.

Não nos cabe, neste editorial, promover uma defesa do uso dos modelos de LLM, mas também não podemos ignorar o impacto dessas tecnologias no cenário atual, especialmente no que tange à sua relação com o processo editorial e o universo das publicações científicas.

Historicamente, uma variedade de ferramentas de IA tem sido utilizada por autores e periódicos para aprimorar a escrita, corrigir a ortografia e a gramática, gerar imagens, detectar plágio e realizar traduções, como é o caso do Google Tradutor, Grammarly, Hemingway Editor, Jasper AI, entre outros ^2^. Com a crescente ampliação do uso dessas plataformas, especialmente entre autores, cabe a nós, editores, e ao mercado editorial, refletir sobre o uso responsável dessas ferramentas, visando otimizar o processo de publicação científica ^3^. Afinal, o problema não reside na tecnologia em si, mas na forma como ela é empregada ^1^.

*Cadernos de Saúde Pública* (CSP) reconhece a necessidade de acompanhar o novo cenário tecnológico e contribuir para o debate e a regulamentação do uso de IA no processo de publicação. Sabemos que o ChatGPT está sendo extensamente utilizado como ferramenta auxiliar à escrita acadêmica. Nesse contexto, nosso objetivo é compreender e orientar o uso desses recursos de maneira transparente e ética, tanto por autores quanto por pareceristas e editores. Essa postura é condizente com a prática de CSP, que é fundamentada nos princípios e diretrizes de integridade em pesquisa recomendadas pelo Comitê de Ética na Publicação (COPE; Committee on Publication Ethics) ^4^.

No que se refere ao trabalho de revisores e editores, a IA pode oferecer suporte em diversos aspectos do processo de publicação. Ferramentas de IA podem ser capazes de identificar, com maior precisão do que as soluções atuais, textos gerados por LLM em comparação àqueles escritos por humanos e os editores podem se beneficiar com essas ferramentas para a manutenção da integridade e do rigor científico. Autores cuja língua principal não é o inglês, por exemplo, muitas vezes são orientados a buscar revisores nativos para aprimorar a linguagem e a gramática. No entanto, os periódicos poderiam sugerir o uso de IA generativa para esse fim, especialmente em casos mais simples ^2^.

Além disso, a IA pode ser empregada para produzir resumos de maior qualidade, prática que os editores podem incentivar, desde que esses processos sejam devidamente creditados no artigo por todas as partes envolvidas ^2^. Em uma fase mais inicial do fluxo do artigo no periódico, os editores podem utilizar essas ferramentas para realizar uma avaliação preliminar do manuscrito, buscando identificar se o tema já foi excessivamente abordado, ou utilizando uma ferramenta de identificação de plágio e conflitos de interesse, para localizar revisores especializados no tema ou para editar a versão aceita para publicação com a finalidade de ajustar o estilo e a linguagem aos padrões da revista ^5^ ^,^ ^6^. Na etapa da padronização e formatação do artigo, como a adaptação dos gráficos e tabelas, a IA pode minimizar tempo e custos da equipe dedicada a essa tarefa, possibilitando que os periódicos com menos recursos financeiros possam otimizar os seus gastos e ampliar a sua longevidade.

CSP considera aceitável que autores utilizem o ChatGPT para auxiliar na estruturação de temas e tópicos de um artigo, funcionando como um rascunho, sugerindo títulos que possam chamar a atenção dos leitores ou frases mais adequadas à linguagem formal, produzindo e corrigindo códigos de programação, auxiliando em análises estatísticas, ou formatando o artigo e as referências conforme as normas da revista. A tradução para outro idioma também pode ser realizada por meio de IA generativa, facilitando a comunicação entre os autores e seus leitores. É fundamental que os autores indiquem, nas seções pertinentes do artigo (como métodos ou agradecimentos), em que etapas a ferramenta foi utilizada. Essas estratégias claras promovem a transparência no uso da tecnologia.

O COPE defende que ferramentas de IA, como o ChatGPT, não podem ser creditadas como autores de artigos científicos, pois não podem assumir responsabilidade pelo conteúdo e nem reivindicar direitos autorais ^4^. CSP apoia essa posição, já que os autores devem aprovar a versão final submetida, garantir sua integridade e assinar termos de concessão de direitos autorais, o que a IA não pode fazer.

Além disso, a redação de artigos científicos exige originalidade, algo impossível para o ChatGPT, uma vez que os textos gerados são apenas uma síntese das proposições mais recorrentes a respeito do assunto solicitado no universo dos dados utilizados em seu treinamento. Isso aumenta o risco de plágio ou alta similaridade entre textos. A IA pode gerar conteúdos muito semelhantes para diferentes artigos, mesmo com variações de forma e estilo, sem a devida atribuição de fontes, o que seria facilmente detectado por softwares de verificação de similaridade. Os conteúdos gerados por IA também estão frequentemente associados a violações de direitos autorais ^2^. Respostas tendenciosas, originalidade limitada e o potencial para disseminar informações incorretas estão entre os principais problemas oriundos de IA generativa, uma vez que os resultados possuem uma confiabilidade reduzida ^7^.

Aplicações da inteligência artificial são disseminadas há anos, antes mesmo que fossem popularmente conhecidas por esse nome, de modo que já alcançaram um estado de ubiquidade no cotidiano da maioria das pessoas. Exemplos disso são os corretores ortográficos de editores de texto, os assistentes de conteúdo de serviços de *streaming* e os filtros de *spam* nos e-mails. Não é de se admirar que justamente pessoas ligadas à pesquisa científica e à inovação estejam mais propensas a experimentar, de maneira consciente e crítica, as possibilidades de uso dessas tecnologias.

A publicação científica precisa acompanhar as transformações contemporâneas. Com uma supervisão rigorosa e uma regulamentação clara e bem definida, o uso de IA no processo editorial pode gerar grandes benefícios. Reconhecer as possibilidades que essa tecnologia oferece já representa um avanço significativo. O grande desafio atual para todos os periódicos é encontrar formas de integrar algumas das tarefas realizadas por IA na rotina diária do processo editorial de maneira prática, eficiente e ética. Desenvolver as melhores práticas e estratégias para uma relação harmoniosa entre IA, periódicos e a publicação científica pode ser o caminho a seguir, gerando benefícios tanto para a ciência quanto para a sociedade como um todo.

# Translation: Inteligencia artificial y proceso editorial en CSP

**DOI:** 10.1590/0102-311XES189024

**Authors:** Luciana Correia Alves, Luciana Dias de Lima, Marilia Sá Carvalho

**Affiliations:** 1 Instituto de Filosofia e Ciências Humanas, Universidade Estadual de Campinas, Campinas, Brasil.; 2 Escola Nacional de Saúde Pública Sergio Arouca, Fundação Oswaldo Cruz, Rio de Janeiro, Brasil.; 3 Programa de Computação Científica, Fundação Oswaldo Cruz, Rio de Janeiro, Brasil.

A lo largo de la historia, los avances tecnológicos siempre han ido acompañados de incertidumbre, como ocurrió en la Revolución Industrial con la aparición de las máquinas en la segunda mitad del siglo XVIII, y en la informática con el desarrollo del sistema 86-DOS en los años 1980. En el arte, la fotografía del siglo XIX inspiró a pintores como Monet y Renoir a desarrollar el impresionismo, incorporando nuevos elementos creativos ^1^. Si bien la capacidad humana para imaginar y crear es esencial, las innovaciones a menudo plantean preocupaciones sobre el potencial de reemplazar la cognición humana.

Ya se han pasado más de 70 años desde que aparecieron las primeras computadoras, y la tecnología que las rodea ha avanzado a pasos agigantados. Con la evolución de la inteligencia artificial (IA), nuevos campos y subcampos han ganado protagonismo, especialmente a partir de noviembre del 2022, cuando las plataformas de IA generativas, incluidos los modelos de lenguaje natural (LLM, por sus siglas en inglés), como el popular ChatGPT, se volvieron ampliamente utilizadas.

No nos corresponde, en este editorial, promover una defensa del uso de los modelos de LLM, pero tampoco podemos ignorar el impacto de estas tecnologías en el panorama actual, especialmente en lo que respecta a su relación con el proceso editorial y el universo de las publicaciones científicas.

Históricamente, una variedad de herramientas de IA ha sido utilizada por autores y revistas para mejorar la escritura, corregir la ortografía y la gramática, generar imágenes, detectar plagio y realizar traducciones, como es el caso de Google Traductor, Grammarly, Hemingway Editor, Jasper AI, entre otras ^2^. Con la creciente expansión del uso de estas plataformas, especialmente entre los autores, nos corresponde a nosotros, los editores y el mercado editorial, reflexionar sobre el uso responsable de estas herramientas, con el objetivo de optimizar el proceso de publicación científica ^3^. Al fin y al cabo, el problema no reside en la tecnología en sí, sino en la forma en que se emplea ^1^.

*Cadernos de Saúde Pública* (CSP) reconoce la necesidad de mantenerse al día con el nuevo panorama tecnológico y contribuir al debate y a la regulación del uso de la IA en el proceso de publicación. Sabemos que ChatGPT se utiliza ampliamente como herramienta auxiliar para la redacción académica. En este contexto, nuestro objetivo es comprender y orientar el uso de estos recursos de manera transparente y ética, tanto por parte de autores como de revisores y editores. Esta postura es consistente con la práctica de CSP, que se basa en los principios y directrices de integridad en la investigación recomendados por el Comité de Ética de Publicaciones (COPE; Committee on Publication Ethics) ^4^.

En lo que respecta al trabajo de revisores y editores, la IA puede ofrecer apoyo en diversos aspectos del proceso de publicación. Las herramientas de IA pueden ser capaces de identificar, con mayor precisión que las soluciones actuales, textos generados por LLM en comparación con los escritos por humanos, y los editores pueden beneficiarse de estas herramientas para mantener la integridad y el rigor científico. Los autores cuyo idioma principal no es el inglés, por ejemplo, a menudo son orientados a buscar correctores nativos para mejorar el lenguaje y la gramática. Sin embargo, las revistas podrían sugerir el uso de IA generativa para este propósito, especialmente en casos más simples ^2^.

Además, la IA se puede emplear para producir resúmenes de mayor calidad, una práctica que los editores pueden fomentar, siempre que todas las partes involucradas acrediten adecuadamente estos procesos en el artículo ^2^. En una etapa anterior del flujo del artículo en la revista, los editores pueden utilizar estas herramientas para realizar una evaluación preliminar del manuscrito, buscando identificar si el tema ya ha sido abordado de manera excesiva, como herramienta para identificar plagio y conflictos de intereses, para ubicar revisores especializados en el tema o para editar la versión aceptada para publicación con el fin de ajustar el estilo y el lenguaje a los estándares de la revista ^5^ ^,^ ^6^. En la etapa de estandarización y formato del artículo, como la adaptación de gráficos y tablas, la IA puede minimizar el tiempo y el costo del equipo dedicado a esta tarea, permitiendo a las revistas con menos recursos económicos optimizar sus gastos y aumentar su longevidad.

CSP considera aceptable que los autores utilicen ChatGPT para ayudar en la estructuración de temas y tópicos de un artículo, funcionando como un borrador, sugiriendo títulos que puedan atraer la atención de los lectores o frases más adecuadas al lenguaje formal, produciendo y corrigiendo códigos de programación, ayudando en análisis estadísticos, o formateando el artículo y las referencias de acuerdo con las normas de la revista. La traducción a otro idioma también se puede realizar mediante IA generativa para facilitar la comunicación entre los autores y sus lectores. Es fundamental que los autores indiquen, en las secciones relevantes del artículo (como métodos o agradecimientos), en qué etapas se utilizó la herramienta. Estas estrategias claras promueven la transparencia en el uso de la tecnología.

COPE sostiene que las herramientas de IA, como ChatGPT, no pueden acreditarse como autores de artículos científicos, ya que no pueden asumir responsabilidad por el contenido ni reivindicar derechos de autor ^4^. CSP apoya esta posición, ya que los autores deben aprobar la versión final enviada, asegurar su integridad y firmar los términos de concesión de derechos de autor, cosa que la IA no puede hacer.

Además, redactar artículos científicos requiere originalidad, algo imposible para ChatGPT, ya que los textos que genera son solo una síntesis de las proposiciones más recurrentes sobre el tema solicitado en el universo de datos utilizados en su entrenamiento. Esto aumenta el riesgo de plagio o alta similitud entre textos. La IA puede generar contenidos muy similares para diferentes artículos, incluso con variaciones de forma y estilo, sin una atribución adecuada de las fuentes, lo que sería fácilmente detectado por un *software* de verificación de similitud. El contenido generado por IA también suele asociarse con violaciones de derechos de autor ^2^. Las respuestas sesgadas, la originalidad limitada y la posibilidad de difundir información incorrecta se encuentran entre los principales problemas que surgen de la IA generativa, ya que los resultados tienen una confiabilidad reducida ^7^.

Las aplicaciones de la inteligencia artificial ya se encuentran muy extendidas desde hace años, incluso antes de que se las conociera popularmente con ese nombre, de modo que ya han alcanzado un estado de ubicuidad en la vida cotidiana de la mayoría de las personas. Ejemplos de esto son los correctores ortográficos en los editores de texto, los asistentes de contenido en los servicios de transmisión y los filtros de *spam* en los correos electrónicos. No es de extrañar que precisamente las personas vinculadas a la investigación científica y a la innovación tengan más probabilidades de experimentar, de manera consciente y crítica, las posibilidades de uso de estas tecnologías.

La publicación científica debe mantenerse al día con las transformaciones contemporáneas. Con una supervisión estricta y una regulación clara y bien definida, el uso de la IA en el proceso editorial puede aportar grandes beneficios. Reconocer las posibilidades que ofrece esta tecnología supone ya un avance significativo. El gran desafío actual para todas las revistas es encontrar formas de integrar, de forma práctica, eficiente y ética, algunas de las tareas realizadas por las IA generativas en la rutina diaria del proceso editorial. Desarrollar las mejores prácticas y estrategias para una relación armoniosa entre IA, revistas y la publicación científica podría ser el camino que seguir para brindar beneficios tanto a la ciencia como a la sociedad en su conjunto.
